# The Pathology of Typhoid Fever

**Published:** 1911-07

**Authors:** E. Bates Block

**Affiliations:** Atlanta, Ga.


### THE PATHOLOGY OF TYPHOID FEVER.

BY E. BATES BLOCK M. D., ATLANTA, GA.

When the tissues of the body become infected with typhoid bacilli its toxins produce local changes in certain organs of the body which are characteristic of the disease, typhoid fever, also by the pyrogenetic action of the toxins fever is produced which leads to changes in the various organs, (cloudy swellings) which is common to all diseases in which there is prolonged high fever and is not specifically connected with the typhoid toxins. Only the former group will be considered in this paper.

THE INTESTINES.

The general surface of the small and large intestines shows catarrhal inflammation with desquamation of epithelial cells.

The most typical lesion of the intestines, however, is the swelling of Peyers patches and the solitary lymph follicles, which may go on to ulceration. The disease affects chiefly the lower part of the ileum especially within a foot of the ileo-caecal valve, occasionally the appendix or colon may show a few patches, more rarely the jejunum or even the duodenum.

The mucus surface becomes swollen and red, covered by an abundant secretion of mucus, the patch is hyperaemic and swollen and remains in this condition for several days. As cell infiltration takes place (about the eighth to the twelfth day) the hyperaemia decreases and disappears; as the blood vessels become compressed the mucous surface becomes paler and the follicles and Peyers patches stand out prominently, are grayish white, and may project three to five mm.

Microscopic examination shows the follicles filled with cells, most of which are mononuclear, well stained, while others are pale and badly stained.

Some of the cells are epithelioid in appearance and contain several nuclei, occasionally cells containing red blood corpuscles are seen.

The infiltration extends to the neighborhood of the follicles, the adjacent mucoso and muscularis; the walls of the blood vessels become infiltrated and compressed causing the whitish appearance. The lymphatic vessels are dilated and filled with cells and show proliferating endothelial cells. The bacilli are found in the follicles, most numerous in the center where the nuclei are palest, also in the lymphatic vessels of the follicles and the submucous tissue. At this stage (10-12 days) resolution may take place, the cells undergo necrosis, or fatty degeneration, disintegrate and become absorbed. But if the blood vessels become choked and the mucous membrane breaks down from coagulation necrosis, then sloughs are formed looking yellowish or grayish, are soft, raised at the edges, and consist of cells, detritus, fibrin, red blood corpuscles, and microorganisms.

When the slough becomes detached, an ulcer is left (third week.) This may be round or oval with the long axis in the long axis of the intestine, opposite the attachment of the mesentery. It may be superficial and affect only the mucosa or it may affect the sub-mucosa also. The necrosis may pass deep into the muscular coat reaching or even perforating the peritoneum—usually the floor is formed by the circular muscular coat. It is usually smooth and clean, sometimes irregular and shreddy, but is not hardened or much infiltrated, the edges are swollen, soft, sometimes congested, not indurated, often undermined and will [illegible]oat when the ulcer is held under water. Around the ulcer and its floor are mononuclear cells and blood corpuscles.

It is very uncommon for an entire Peyers patch to slough away. A large patch may show three or four ulcers.

Healing of the ulcer takes place by cicatrization in case of recovery. This is effected by granulation and formation of fibrous itssue, the mucous membrane growing in from the edges and the glandular elements may become regenerated to some extent, but the healed ulcer is somewhat depressed and usually pigmented.

Perforation is most common in the ileum, next in the appendix or colon—appendicitis and typhoid fever have often been associated.

In the experimental production of Typhoid fever in the higher monkeys, Metchinkoff observed swelling and hyperaemia of Peyers patches but never any ulceration. The mesenteric glands were hypertrophied, often congested, and contained necrotic masses and typhoid bacilli.

In man the mesenteric glands become enlarged by hyperaemia, especially in their peripheral parts, and as they swell more become paler and softer. Spots of necrosis are common. The infiltrated cells undergo fatty degeneration, and become absorbed and then the gland gradually diminishes in size and becomes firmer. Occasionally it may undergo suppuration and may rupture into the peritoneal cavity and cause peritonitis, or it may become inspissated and eventually be calcified. They are most affected in the mesentery near the lower part of the ileum. They contain many typhoid bacilli and their overflow into the circulation produces a bacillaemia but not a true septicaemia. The retroperitoneal glands are also swollen.

### Spleen.

The spleen gradually increases in volume and by the end of the third week may be three or four times its normal size and weight. The swelling is less marked in old people.

First, there is hyperaemia and on section the spleen looks cherry red in color, and the stroma is indistinct; the pulp becomes softer, bulges forward; the Malpighian bodies are more distinct and larger (third weeks). In the fourth and fifth week the spleen becomes smaller, the pulp appears pale or brownish, the consistence increases and the stroma more fibrous. At the height of the disease there is an infiltration of leucocytes, some of them degnerated, large endothelial cells with one or more nuclei, and large cells containing one or more red blood corpuscles. The B. Typhosis are found often in clumps. Intarcts or abscesses may occur.

The tonsils and glands of the pharynx are enlarged and infiltrated.

Rieser reported two cases of laryngeal inflammation, and Boston has reported oedema of the glottis and infiltration of the larynx.

The nudulla of the bones undergo much the same changes as the spleen.

The stomach often shows catarrhal inflammation and the adenoid tissue may show cellular infiltration.

Wichern has reported two cases of dilatation of the stomach.

THE KIDNEYS.

The so-called lymphomata of the kidneys have not been fully studied but it seems quite probable that they may be of the same nature as the typhoid nodules in the liver.

They usually show cloudy swelling of the epithelium of the convoluted tubules. They are enlarged, especially the cortex, and the epithelium of the convoluted tubules appear granular. Perivascular cell infiltration may also be noted. Small cell infiltration may occur around the blood vessels.

Brownlee reported hemorrhages into the tubules due to destruction of the basement membrane, with small interstitial hemorrhages.

Cagnetta and Zancan made experiments of injecting B. Typhosis and its toxins in animals and found degenerative phenomnea especially in the secretory epithelium of the contorted tubules. Also exudative and proliferative appearances and severe disturbances of the circulation, hemorrhages between the uriniferous tubules, oedema and small cell infiltration of the stroma, later organization of a small part of the cells of the infiltration.

The so-called lymphomata in Typhoid kidneys is not constant and is not dependent on the presence of the bacilli, but may be just as easily produced by their products.

THE SKIN.

Philipovicz's sign or the orange yellow discoloration which appears on the palms of the hands and soles of the feet is often followed by desquamation and is often associated with a diminution or abolition of tactile sensibility, and is supposed to be due to trophic alteration of the epidermis from the toxins being eliminated through the skin

Appiani and others have reported cases of general desquamation of the skin in typhoid convalescence which is a trophic occurrence like the changes in the nails and falling out of the hair.

Rarely gangrene may occur and this is due to thrombosis and may affect any part of the body.

There has been no satisfactory histological work done on the nose spots, but they are due, as far as our knowledge goes, to simple hyperaemia due to the presence in them of typhoid bacilli.

The gall bladder may be inflamed, filled with grey or light colored bile, and rarely there may be suppuration, ulceration and perforation of the gall bladder. More often the catarrh of the gall bladder leads to the formation of gall stones.

LIVER.

The so-called lymphoid nodules which appear in the liver have been shown to have nothing at all to do with lymphoid tissue, but are due to focal cell necrosis. They may occupy the neighborhood of the portal or hepatic veins or a whole lobule may undergo necrosis. They are round, oval, or irregular in shape and are rarely if at all visible to the unaided eye. At first the nuclei of the liver cells become pale and swollen and then the nucleus undergoes disintegration or absorption, the protoplasm does not stain weel, takes a red stain with eosin and methylene blue. Polynuclear leucocytes wander into the cell body, the outlines of the cell becomes indistinct, it disintegrates, they disappear and are replaced by numerous small, round, deeply stained nuclei mixed with few oval or spindle shaped nuclei, which marks the beginning of connective tissue formation with healing by scar formation, in which blood vessels and bile ducts may be reproduced.

The capillaries are sometimes dilated and contain large numbers of typhoid bacilli, clumps of which often grow post-mortem. Acute yellow atrophy or abscess may rarely occur.

The muscles may show hyaline or fatty degeneration or proliferation of the muscle nuclei.

Guillain has described idiopathic muscular atrophy following typhoid fever, and cases of pseudo-hypertrophic muscular paralysis have been reported.

### The Heart.

The heart is flabbly, pale, soft, sometimes the muscle fibres show fatty degeneration and occasionally waxy degeneration, and in some cases of sudden death segmentation of the mucular elements has been found. Occasionally there is cell infiltration between the fibres and the small arteries of the myocardium may show endarteritis. Endocarditis or pericarditis may occur.

### The Lungs.

The lungs may show hemorrhagic infarcts, gangrene or abscesses. Bronchopneumonia or lobar pneumonia may occur, and in the latter case when due to the typhoid bacillus the area assumes a markedly hemorrhagic type.

The nervous system may show oedema of the membranes of the brain, distension of the ventricles, pigmentation of the ganglion cells, infiltration of the perivascular spaces with leucocytes, also the spaces around the ganglion cells.

There may be fatty degeneration of nerve fibres and hemorrhages. Rarely meningitis occurs.

Peripheral neuritis is not uncommon and is the cause of tender toes and sometimes paralysis. Slight and transitory alteration of the cortical nerve cells by the typhoid toxins, may give rise to Jacksonian epilepsy, hemiplegia, or asphasia, while in other cases these results may be brought about by thrombosis of the cerebral arteries or hemorrhage.

Giacomucci has reported convulsions and asphasia with paramyonclous multiplex in two cases.

The occurrence of typhoid bacilli in colonies or clumps in the blood vessels or tissues is usually due to their multiplication after death, and it is very improbable that they are the cause of thrombosis of blood vessels or the cause of necrosis in the tissues, as the latter can be produced by the injection of the toxins alone, while the former is equally common in other febrile diseases with diarrhoea and thickening of the blood.

It seems from the study of the pathology of typhoid fever that the typhoid bacillus or its toxins possess ordinarily a greater capacity for producing necrosis than pus formation and the former is the lesion usually found. The frequency with which the B. Typhosis has been found associated with other micro-organisms in pus formation has led to the hypothesis that the pus formation is in reality due to the secondary organisms, and in the cases where the typhoid bacillus has been isolated in pure cultures it has been supposed by many writers that the secondary organism after producing pus formation has died out, leaving only the B. Typhosus. On the other hand, the examples of pus formation are fairly numberous where the B. Typhosus was obtained in pure culture from various organs in the body.

